# Novel Hepatitis E Virus Genotype in Norway Rats, Germany

**DOI:** 10.3201/eid1710.101399

**Published:** 2011-10

**Authors:** Wen Zhang, Quan Shen, Xiuguo Hua, Li Cui

**Affiliations:** Jiangsu University, Jiangsu, People’s Republic of China (W. Zhang);; The Ohio State University, Wooster, Ohio, USA (Q. Shen);; Shanghai JiaoTong University, Shanghai, People’s Republic of China (X. Hua, L. Cui)

**Keywords:** hepatitis E virus, viruses, Germany, rats, letter, genotype, Norway, HEV

**To the Editor:** We read with interest the article by Johne et al. about 2 novel hepatitis E virus (HEV) isolates in Norway rats in Germany (*1*). Some points in the report deserve comment.

First, because of degeneracy of the genetic code, HEV amino acid sequences are more conserved than nucleotide sequences. For instance, although the open reading frame 2 of the avian HEV isolate (GenBank accession no. AY535004) has only 65% nt sequence homology to that of the swine HEV isolate swGX32 (GenBank accession no. EU366959), their amino acid sequences shared >90% identity. However, the table in (*1*) indicated the amino acid sequence homologies between the novel and previous HEV isolates were similar to (some even lower than) the nucleotide sequence homologies. These low sequence identities of the capsid proteins between the novel and previous HEVs may explain why no HEV antibody–positive rat was found in the initial serologic screening with a commercial genotype 1–based ELISA. Furthermore, we wonder how the novel antigen in the hepatocytes could react with the anti-HEV serum in the immunohistochemical staining.

Second, the authors stated they determined the entire virus genome by using a previously described method (*2*). The primers in that method were designed to amplify a genotype 3 HEV isolate with low (55.7%) sequence homology to the 2 novel HEV isolates and therefore cannot amplify their sequences. We ask the authors to list the new primer sequences they used, which will help determine the full viral genome if this virus is found in other regions or animal species.

Suggesting the rabbit HEV sequences may be representative genotype 3 isolates is not yet appropriate because not enough research has yet determined whether rabbit HEV infects other species. Therefore, the rabbit HEV sequence FJ906895 should not be listed as representative genotype 3 isolate as in Figure 1 in (*1*). Also, the swine isolate DQ450072 should not be listed as a representative genotype 4 isolate; a recent report indicated it was a recombinant produced between genotypes 3 and 4 isolates (*3*).
