# Supplementary material for: Novel Hepatitis E Virus Genotype in Norway Rats, Germany
Source: Emerg Infect Dis. 2011 Oct;17(10):1981–2. doi: 10.3201/eid1710.101399 (PMC3310647; doi:10.3201/eid1710.101399)
Supplement: Related texts

# Reply

**Authors:** Reimar Johne, Gerald Heckel, Paul Dremsek, Anita Plenge-Bönig, Eveline Kindler, Christina Maresch, Jochen Reetz, Anika Schielke, Rainer G. Ulrich

**Affiliations:** Federal Institute for Risk Assessment, Berlin, Germany (R. Johne, J. Reetz, A. Schielke);; Swiss Institute of Bioinformatics, Genopode, Lausanne, Switzerland (G. Heckel);; University of Bern, Bern, Switzerland (G. Heckel, E. Kindler);; Friedrich-Loeffler-Institut, Greifswald–Insel Riems, Germany (P. Dremsek, C. Maresch, R.G. Ulrich);; Institute of Hygiene and Environment Hamburg, Hamburg, Germany (A. Plenge-Bönig);; Free University of Berlin, Berlin, Germany (A. Schielke)

**In Response**: The major objective of our study was determination and initial characterization of the entire nucleotide sequence of a novel hepatitis E virus (HEV) from Norway rats. We demonstrated high levels of nucleotide and amino acid sequence divergence between HEV strains from the novel rat and other mammalian and avian HEV strains. In line with our data, nucleotide (and amino acid) sequence identities of 50%–53% (42%–49%), 51%–57% (42%–55%), and 45%–46% (20%–29%) were reported for open reading frame (ORF) 1, ORF-2, and ORF-3, respectively, between rat, human, and avian HEV strains (*1*). Of course, the genetic code is degenerated; however, no strict relationship exists between divergence in nucleotide and corresponding amino acid sequences, e.g., because of natural selection processes (*2*). We could not reproduce the high level (>90%) of amino acid sequence identity between the capsid protein (CP) of avian HEV (GenBank accession no. AY535004) and the unpublished GenBank entry swGX32 (accession no. EU366959) claimed by Zhang et al. (*3*).

The low level of amino acid sequence identity between rat and human HEV strains might explain the lack of reactivity of transudates from 6 investigated rats in the genotype 1–based ELISA. Consistent with this assumption, a rat hyperimmune serum specimen, raised against a truncated recombinant rat HEV CP derivative, reacted strongly with the homologous antigen but weakly with genotype 3 HEV antigen (P. Dremsek and R.G. Ulrich, unpub. data). Nevertheless, conserved and cross-reactive epitopes have been identified in the CP of HEV (*4*) and can be expected in the antigenic protruding domain of rat HEV CP (*5*). Therefore, some cross-reacting antibodies might exist that would explain detection of rat HEV by the human anti-HEV serum used in immunohistochemical staining.

For sequencing, novel primers were designed (Table). The recombinant nature of strain DQ450072 was not known at time of analysis. Nevertheless, that this sequence clusters near, but not within, the genotype 4 branch is consistent with the reported recombination event.

**Table:** Primers used in study (*1*) of amplification of complete genome sequence of rat HEV from rat sample 63, Germany*

Designation	Binding position	Sequence† (5′ → 3′)
rHEV-RACEn‡	307–284	GTGCTCATTAATAGATCGAGGGTG
rHEV-RACE‡	336–313	GGAAGAAAACATCTGTGAATGACA
HEV-100s	78–102	CGGCCAATTCCGCCYTGGSRAATGC
rHEV-900as	905–881	TATGCCCGCCCGCACTAAACAAACT
HEV-800s	776–801	GTGCGGGCCATTGGCTGYCAYTTTGT
rHEV3300as	3099–3074	AGCCGCCATTCTGTTGGCTCCAGATT
rat5-s	2921–2943	CGCCGGGTTGTGATKGAYGAGGC
rHEV-td1-as	4062–4037	GAAATGCCCTGCCCGACCTTGCCATG
HEV-cs	3977–3999	TCGCGCATCACMTTYTTCCARAA
HEV-cas	4446–4424	GCCATGTTCCAGACDGTRTTCCA
Rat HEV-inv-s	4301–4322	GGGGCRCCYGAGTGGATGTGGA
63-5400as	5449–5428	CTCAGTCGCCATGATATGCGTA
#8-ORF2-s	5399–5421	CCCTTACTGCCTYTKCAGGAYGG
#8-ORF2-as	5604–5582	GTGGAAGTGATGGAATTCATRTC
63-5500s§	5555–5576	CAATCCACAACAGTCCCCACGT

*HEV, hepatitis E virus; RACE, rapid amplification of cDNA ends; ORF, open reading frame. †D = A + G + T; K = G + T ; M = A + C; R = A + G; S = G + C; Y = C + T. ‡Primers were used for 5′-RACE. §Primer was used for 3′-RACE.

Virus taxonomy has to “categorize the multitude of known viruses into a single classification scheme that reflects their evolutionary relationships” (*6*). Because the evolutionary relationships could not be determined without sequence analyses, we could not follow the suggestion of Zhang et al. to use other than genetic information for genotype classification (*3*). Future classification of HEV strains would profit from definition of solid criteria and distinct thresholds for definition of genotypes.

## References

[1] Batts W, Yun S, Hedrick R, Winton J. A novel member of the family *Hepeviridae* from cutthroat trout (*Oncorhynchus clarkii*). Virus Res. 2011;158:116–23. 10.1016/j.virusres.2011.03.01921458509

[2] Holmes EC. The evolution and emergence of RNA viruses. Oxford series in ecology and evolution. Oxford (UK): Oxford University Press; 2009.

[3] Zhang W, Shen Q, Hua X, Cui L. Novel hepatitis E virus genome in Norway rats, Germany. Emerg Infect Dis. 2011;17:1981–2.2200039710.3201/eid1710.101399PMC3310647

[4] Haqshenas G, Huang FF, Fenaux M, Guenette DK, Pierson FW, Larsen CT, The putative capsid protein of the newly identified avian hepatitis E virus shares antigenic epitopes with that of swine and human hepatitis E viruses and chicken big liver and spleen disease virus. J Gen Virol. 2002;83:2201–9.1218527410.1099/0022-1317-83-9-2201

[5] Johne R, Plenge-Bönig A, Hess M, Ulrich RG, Reetz J, Schielke A. Detection of a novel hepatitis E-like virus in faeces of wild rats using a nested broad-spectrum RT-PCR. J Gen Virol. 2010;91:750–8. 10.1099/vir.0.016584-019889929

[6] Ball LA. The universal taxonomy of viruses in theory and practice. In: Fauquet CM, Mayo MA, Maniloff J, Desselberger U, Ball LA, editors. Virus taxonomy. Eighth report of the ICTV. San Diego (CA): Elsevier Academic Press; 2005. p. 3–8.
